# Determining an optimal pool size for testing beef herds for Johne’s disease in Australia

**DOI:** 10.1371/journal.pone.0225524

**Published:** 2019-11-20

**Authors:** Anna Ly, Navneet K. Dhand, Evan S. G. Sergeant, Ian Marsh, Karren M. Plain

**Affiliations:** 1 Faculty of Science, Sydney School of Veterinary Science, The University of Sydney, Camden, NSW, Australia; 2 Ausvet Pty Ltd., Canberra, Australia; 3 Elizabeth Macarthur Agricultural Institute, NSW Department of Primary Industries, Menangle, Australia; Faculte de medecine veterinaire, Universite de Montreal, CANADA

## Abstract

Bovine Johne’s disease (JD) is a chronic debilitating disease affecting cattle breeds worldwide. Pooled faecal samples are routinely tested by culture to detect *Mycobacterium avium* subsp. *paratuberculosis* (*Mptb*) infection. More recently, a direct high throughput molecular test has been introduced in Australia for the detection of *Mptb* in faeces to circumvent the long culture times, however, the optimal pool size for beef cattle faeces is not known. This study aimed to determine the optimal pool size to achieve the highest test sensitivity and specificity for beef cattle. Individual archived faecal samples with low, medium and high quantities of *Mptb* (n = 30) were pooled with faecal samples from confirmed JD negative animals to create pool sizes of 5, 10, 15 and 20, to assess the diagnostic sensitivity relative to individual faecal qPCR. Samples from JD-free cattle (n = 10) were similarly evaluated for diagnostic specificity. Overall, 160 pools were created, with *Mptb* DNA extracted using magnetic bead isolation method prior to *Mptb*-specific IS*900* quantitative PCR (qPCR). The pool size of 10 yielded the highest sensitivity 73% (95% CI: 54–88%), regardless of the quantity of *Mptb* DNA present in the faeces. There was no significant differences between the four different pool sizes for positive pool detection, however, there was statistical significance between low, medium and high quantities of *Mptb*. Diagnostic specificity was determined to be 100%. The increase in pool size greater than 10 increased the chances of PCR inhibition, which was successfully relieved with the process of DNA dilution. The results of this study demonstrate that the pool size of 10 performed optimally in the direct faecal qPCR. The results from this study can be applied in future simulation modelling studies to provide suggestions on the cost-effective testing for JD in beef cattle.

## Introduction

Bovine Johne’s disease (JD) is a chronic disease affecting ruminant species worldwide, caused by *Mycobacterium avium* subsp. *paratuberculosis* (*Mptb*). This disease causes granulomatous enteritis, resulting in major productivity losses for the animal. The most common transmission pathway for *Mptb* is the faecal-oral route. An animal becomes infected by grazing and ingesting *Mptb* from the faeces of infected animals. Subclinically infected animals intermittently shed *Mptb* in their faeces and both the quantity and consistency of shedding increases as the animals become clinically diseased. Clinical signs in infected cattle can occur by 2–4 years of age. Clinically affected JD cattle may display progressive emaciation and develop chronic intermittent diarrhoea. These clinically affected animals may excrete up to 5 x 10^12^ *Mptb* bacilli per day [1–5].

In Australia, JD has predominately been managed with geographical disease zoning [6]. During 2015, Animal Health Australia undertook a review of the National JD Strategic Plan 2012–20 and prepared a revised plan in consultation with Australian cattle industry bodies and other stakeholders. This revised approach recognises the role of the producer as a decision-maker, requiring no State or Territory regulation to manage the disease and imposing no regulated transactional or movement restrictions on producers [7]. This has put the onus on beef producers to maintain a disease-free herd or to improve the Johne’s Beef Assurance Scheme (J-BAS) score of their herd [8]. The J-BAS score provides information about the risk of JD being present on a property and can be used by producers to indicate their property status or assess the risk of purchasing a JD infected animal.

To achieve and maintain a high J-BAS score, producers must demonstrate an absence of *Mptb* infection on the property in the past five years, prepare and implement a biosecurity plan, and have their animals regularly tested [8]. The current testing protocol for J-BAS requires conducting two tests: (a) an initial Sample test to screen a representative sample (up to 300) of the adult herd (or the whole herd for properties with <300 cattle) and (b) a triennial Check test of 50 if the most susceptible adult animals in the herd. Although a number of tests including ELISA and pooled faecal culture (PFC) have been approved for use, the pooled High-Throughput–Johne’s (HT-J) faecal quantitative PCR (qPCR) is considered to be the test of choice as it has a higher sensitivity and specificity than the ELISA and a relatively fast turnaround compared to faecal culture [9–11]. Moreover, it has been validated in the Australian context [9].

The aim of pooling faecal samples for diagnostic application is to minimise the costs without jeopardizing diagnostic sensitivity and specificity [12]. Pooling of faecal samples allows producers to know the infection status of the herd, as more individual animals tested in pools, may only marginally increase the laboratory costs, compared to individual animal testing [12–18]. However, a common issue associated with the pooling of faecal samples is the presence of inhibitory substances that could interfere with PCR amplification [19]. PCR inhibitors may arise due to the complex nature of the faecal matrix of ruminants, with a variety of endogenous (high diversity of bacterial species) and exogenous (herbivorous matter) components [20, 21]. This can be detrimental for infection detection, especially for paratuberculosis, as false negative results can lead to the introduction of infected animals into disease-free herds. Strategies to remove inhibitors from faecal samples include removing the inhibiting agents directly or the dilution of undiluted DNA extract [19, 22]. The addition of either strategy requires additional laboratory processing steps, which may reduce the availability of target amplicon concentrate in the extract.

This is especially problematic for animals that are low *Mptb* shedders, as the reduction of analyte concentration present in the sample, leads to the ‘dilution’ effect. From this, there would be greater stochasticity in the sampling probability of analyte detection, particularly after the dilution of the samples, ultimately leading to a reduction in sensitivity [23].

Currently, a pool size of 5 is used for the testing of cattle faeces by qPCR for *Mptb* detection [24]. Previous studies in dairy cattle using PFC had demonstrated that the small pool size of 5 had greater sensitivity, compared to larger pool sizes [12, 16, 25, 26]. Based on PFC results alone, it has been recommended that the same pool size of 5 be applied to the HT-J qPCR for J-BAS testing. However, when using the pool size of 5 with the HT-J qPCR to obtain J-BAS scores, producers may have to pay approximately $7200 AUD for the initial Sample tests, followed by an additional $1200 AUD for the triennial Check test, with prices dependent on individual laboratory charges (pers. comm.). This high cost associated with the current pooling regime puts a significant economic burden on the beef industry and may discourage producer participation in the program.

The objective of this study was to investigate alternative pooling strategies to facilitate more cost-effective herd-level testing for JD, while maintaining test sensitivity and specificity and managing PCR inhibition. A validated faecal qPCR test for individual faecal samples was applied to pooled samples, with the quantity of DNA detected and number of positive results compared. An investigation on the dilution of the undiluted DNA extract was also conducted to determine whether inhibiting agents were mitigated in the samples.

## Materials and methods

### Selection and characteristics of cattle faecal samples

Sample size was calculated by simulation. Assuming that the sensitivity estimates of pooled testing relative to individual faecal testing for pool sizes of 5, 10, 15 and 20 were 65%, 60%, 35% and 30%, respectively, a sample size of 28 pools for each group (i.e. a total sample size of 120 assuming equal group sizes) was required to achieve a power of 80% for detecting a difference in sensitivity at a two-sided chi-square test p-value of 0.05. We decided to use a sample size of 30 to ensure selection of 10 samples from each of the low, medium and high *Mptb* faecal shedding levels.

Archived faecal samples used in this study were collected from naturally infected cattle or previous cattle trials conducted by the Farm Animal Health group at the University of Sydney, Camden [9]. The archived faecal samples were stored at -80°C (seven years for for *Mptb* positive and up to 10 years for *Mptb* negative samples) prior to processing for this study. All animal experiments were approved by the University of Sydney Animal Ethics Committee.

The negative faecal samples used in this project were from samples collected from control-unexposed cattle during longitudinal animal trials performed at the University of Sydney, Camden [27]. The individual negative faecal samples (n = 10), derived from cattle from a single unexposed herd, were used for the testing of specificity, with additional bulk negative faecal samples (n = 2) used for the creation of pools. All negative faecal samples were negative in the individual *Mptb* faecal qPCR (HT-J test) and faecal culture tests [9, 27]. The positive archived faecal samples used in this study were from naturally infected beef cattle from a herd in Tasmania, Australia. The samples had previously tested positive in an individual HT-J qPCR test.

**Fig 1:**
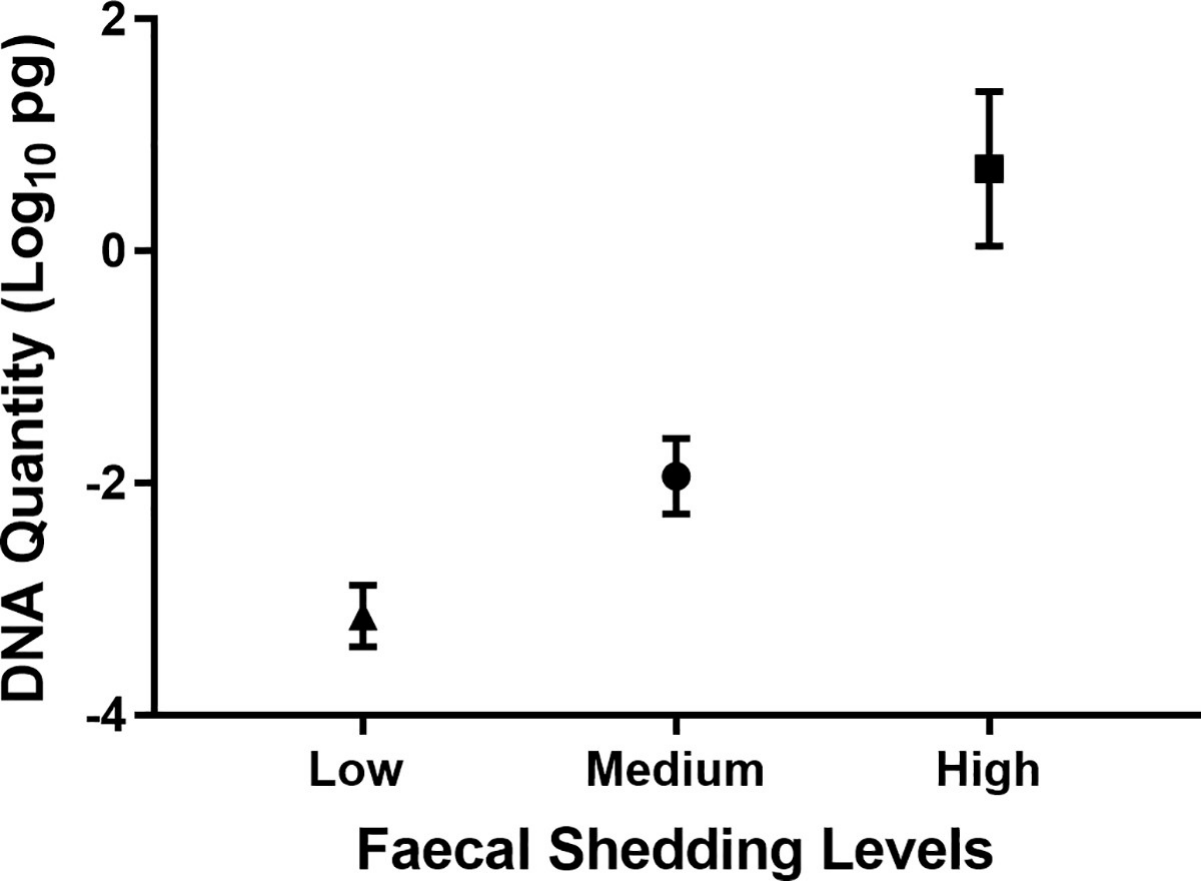
Mean (±SD) DNA quantities of the positive faecal cattle samples detected by individual HT-J qPCR test for the Low (n = 10), Medium (n = 10) and High (n = 10) groups. The cut-point for a positive individual HT-J is 0.001 pg (-3 on the above scale).

Samples were selected to represent the different shedding levels previously observed with field samples, tested with individual HT-J qPCR test [9]. This was informed from the range of HT-J qPCR test results identified during the validation of the HT-J qPCR method on Australian cattle [9]. The positive faecal samples, collected from cattle from a single endemically infected herd, were categorised into Low, Medium and High faecal shedding groups based on the DNA quantity detected in individual HT-J qPCR results. For the low shedders group (n = 10), the *Mptb* DNA quantity detected in the qPCR test was between 0.0005–0.005 pg, the medium group (n = 10) had *Mptb* DNA quantity between 0.005–0.1 pg, and the high group (n = 10) had *Mptb* DNA quantity between 0.1–10 pg (Fig 1).

Not all samples selected were *Mptb* faecal culture positive; as per the validation of the HT-J test, more samples were test positive using the HT-J test than in faecal culture. This was particularly evident at the lower DNA quantity ranges, as the low, medium and high group, had 3, 8 and 10 samples culture positive, respectively [9].

### Pooling of samples

Individual positive (n = 30) and negative samples (n = 10) were pooled into pool sizes of 5, 10, 15, and 20. Each pool contained faeces from individual positive or negative samples and bulk negative faeces from two animals, with pooling ratios at 1:4, 1:9. 1:14 and 1:19, by weight. For this study, 120 pools were created from positive samples for the estimation of sensitivity and 40 pools were created from negative samples for the estimation of specificity (Table 1). A pool size is the number of animals from which faecal samples were pooled. For example, for a pool size of 10, faeces were pooled from 1 positive animal (1 g) and the proportional contribution by weight of faeces from 9 negative animals (1 g each).

**Table 1:** The pooling ratio of positive and negative faeces for each pool size tested.

Pool size	Pool creation			Test input	Pools tested		
	Positive faeces (g)	Negative faeces (g)	Saline (ml)	Amount (g)	Number of positive pools tested	Number of negative pools tested	Total pools tested
**5**	2	8	10	4 +/- 0.3	30	10	40
**10**	1	9	5	3 +/- 0.3	30	10	40
**15**	1	14	7.5	4 +/- 0.3	30	10	40
**20**	1	19	10	3 +/- 0.3	30	10	40

The pooled faecal samples were blended with saline to create a homogenised mixture. The faecal samples were homogenised with an Interscience MiniMix blender for 60 s, before the homogenised faecal samples were transferred to a sterile 25 mL tube.

### Pooled test

Following blending of the pooled faeces, 3 or 4 ± 0.3 g of homogenised faeces (Table 1) was added to 10 mL of sterile saline in a 15 mL sterile tube. The faecal suspension was mixed for 5 s and allowed to settle for 5 min, before inverting the tube to dislodge floating debris. The suspension was allowed to settle for 30 min, followed by the transfer of 3–5 mL of the supernatant to a 15 mL sterile centrifuge tube. The supernatant was centrifuged at 900 x *g* for 30 min to obtain a pellet. The supernatant was then discarded, and 600 μL of lysis buffer (BioSprint 96 one-for-all vet kit; Qiagen– 597.2 μL buffer RLT and 2.8 μL carrier RNA reconstituted in buffer AVE) was added to the pellet. The lysis and pellet suspension was then transferred into 2.0 mL screw cap bead tubes, containing 0.3 g of 0.1 mm diameter zirconia/silica beads for mechanical bead beating.

Bead beating and magnetic bead-based DNA purification were performed as previously described by Plain et al. (2014). Briefly, the suspension was mechanically disrupted with a TissueLyser II (Qiagen), centrifuged at 16,000 x *g* for 3 min in a microfuge, and 500 μL of the supernatant transferred to a sterile 1.5 mL tube and the centrifugation repeated. The DNA purification was conducted with the BioSprint 96 One-for-all Vet kit (Qiagen) using 96-well plates and an automated magnetic particle processor (MagMax Express 96; Life Technologies). A process control (buffers alone) and *Mptb* positive control (5.67 x 10^3^ *Mptb*/well) were included during the DNA purification step.

The eluted DNA extracts were further diluted five-fold and 25-fold; the undiluted, five-fold and 25-fold dilutions were assessed using qPCR. This was based on the recommendations from a previous study, which investigated PCR inhibition in cattle faecal samples, in which dilution of the DNA extract increased the overall sensitivity of the individual HT-J qPCR from 55 to 80% relative to faecal culture [22]. Dilutions were performed automatically with AVE buffer (RNase free water with 0.04% sodium-azide, Qiagen) using the QIAgility (Qiagen). The undiluted and five-fold DNA dilutions were tested in duplicate qPCR reactions, while the 25-fold DNA dilution was tested in a single qPCR replicate. The present study also included a 10-, 15- and 20-fold dilution of the DNA extracts for the faecal pool size 10 samples to determine the optimal dilution to relieve PCR inhibitors.

An IS*900* qPCR was performed to detect *Mptb* DNA using Mx3000P real time PCR instrument (Stategene, Agilent) [9, 28]. Each qPCR reaction had a total volume of 25 μL; 5 μL of DNA template, 250 nM each of forward and reverse primers (MP10-1 5’-ATGCGCC ACGACTTGCAGCCT-3’ and MP11-1 5’-GGCACGG CTCTTGTTGTAGTCG-3’), 12.5 μL of SensiMix SYBR Low ROX qPCR mastermix (Bioline) and 7.4 μl of nuclease free water. The reaction parameters were: an initial denaturation at 95°C for 8 ½ min, 40 cycles of denaturation at 95°C for 30 s and annealing/extension at 68°C for 60 s, followed by melt curve analysis from 65 to 95°C. *Mptb* DNA quantification was conducted with reference to a standard curve included in every qPCR experiment, comprising a 10-fold serial dilution of *Mptb* genomic DNA ranging from 10–0.001 pg/reaction.

The criteria for a positive IS*900* qPCR amplification was an amplification curve with Tm range of 89.1 ± 1.5°C. The acceptance criteria for each IS*900* qPCR experiment was: (i) an amplification efficiency for the *Mptb* genomic DNA standard curve of between 90–110% for at least 4 of the 5 standards, with at least one of the standard 5 (0.001pg) IS*900* qPCR replicates having positive amplification, and (ii) a negative result (no positive amplification curve) for the no template control. The acceptance criteria for each 96 well qPCR extraction plate was: (i) the positive faecal control was positive in the IS*900* qPCR (replicates with positive amplification curves), and (ii) the negative process control had a negative result in the IS*900* qPCR (no positive amplification curve).

To be classified as a HT-J positive result, a sample had to meet the following criteria: (i) the HT-J extraction plate and IS*900* qPCR experiments including this sample passed the above acceptance criteria (ii) the average DNA quantity of the positive IS*900* qPCR sample replicates exceeded the ≥ 0.001 pg *Mptb* genomic DNA cut point. A sample was considered positive if the undiluted or diluted DNA extract was HT-J positive.

### Statistical analysis

The DNA quantity cut-off point was determined with the complete dataset using ROC curve analysis [29]. For a given pool size and dilution, DNA quantity results from positive and negative pools were stacked to conduct ROC curve analysis. The cut-off was selected to maximise sensitivity when the specificity was perfect. The cut-off point of 0.001pg of *Mptb* genomic DNA is in agreement with a previous study [9]. The HT-J results of the difference between the original DNA quantity and that of the five-fold dilution for pool size 10 was compared using a Bland Altman plot from the BlandAltmanLeh package on R Studio [30]. This analysis estimates the difference in the average HT-J qPCR DNA quantities to allow for an approximation of the overall difference between the two DNA quantities.

Test sensitivity, relative to individual faecal qPCR results was calculated as the proportion of the tested pools for a particular pool size within a shedding group that tested positive. Exact 95% confidence intervals (CI) for test sensitivity and specificity were estimated using the FREQ procedure in SAS 9.4 (SAS Institute Inc., Cary, N.C., USA). Sensitivity was compared between pool sizes using generalised linear mixed models fitted using the GLIMMIX procedure in SAS by including the positive/negative status of the pools as an outcome; the pool size (5, 10, 15 or 20), the *Mptb* shedding level (low, medium or high) and their interaction as fixed effects; and the unique animal identification number for the positive animal included in the pool as a random effect. Statistical significance of the variables was evaluated using chi-square tests. Goodness-of-fit of the final model was evaluated using Hosmer and Lemeshow goodness-of-fit test in the LOGISTIC procedure without the random effect as this functionality is not available in the GLIMMIX procedure. A total of 120 observations representing 40 pools each for low, medium and high *Mptb* shedding levels were included in this analysis. The test specificity was estimated as the proportion of the negative pools of a particular size that tested negative.

## Results

### Detection of positive pooled samples in different shedding groups with undiluted and diluted DNA extract

The proportion of positive pools in different pool sizes are shown in Table 2. The pool size of 10 had the highest number of positive results detected by qPCR in the low group, with 3/10 detected when the DNA extract was diluted five-fold (Table 2). Likewise, for the medium group, a pool size of 10 detected 9/10 positives, overall, in comparison to pool sizes 5, 15 and 20, at 7/10, 5/10 and 7/10 positive results, respectively. Despite pool size 5 detecting more positive results (5/10) for the undiluted DNA extract compared to pool size 10 (3/10), the process of dilution led to a greater detection rate in pool size 10 (7/10). When the results were considered for both the undiluted and five-fold dilution, the pool size of 10 (9/10) led to the detection of the most positive samples.

**Table 2:** Number and proportion of HT-J positive results detected at each pooling rate and for each dilution of the DNA extract.

*Mycobacterium avium* subsp. *paratuberculosis* faecal shedding level	DNA Dilution	Proportion of positive pools (Number of HT-J positive pools (%) out of 10 pools)			
		5	10	15	20
**Low**	**Undiluted**	1/10	1/10	0/10^a^	0/10^a^
	**1 in 5**	2/10	3/10	2/10	0/10
** **	**1 in 25**	1/10	2/10	2/10	2/10
	**Any**^b^	**2/10**	**3/10**	**2/10**	**2/10**
**Medium**					
** **	**Undiluted**	5/10	3/10	0/10^a^	0/10^a^
** **	**1 in 5**	6/10	7/10	5/10	7/10
** **	**1 in 25**	3/10	4/10	4/10	4/10
	**Any**^b^	**7/10**	**9/10**	**5/10**	**7/10**
**High**					
** **	**Undiluted**	8/10	8/10	0/10^a^	0/10^a^
** **	**1 in 5**	10/10	10/10	10/10	10/10
** **	**1 in 25**	10/10	10/10	10/10	10/10
** **	**Any**^b^	**10/10**	**10/10**	**10/10**	**10/10**

^a^No positives detected due to PCR inhibition.

^b^Any is HT-J positive in any of the DNA dilutions tested for a particular pool size.

**Fig 2:**
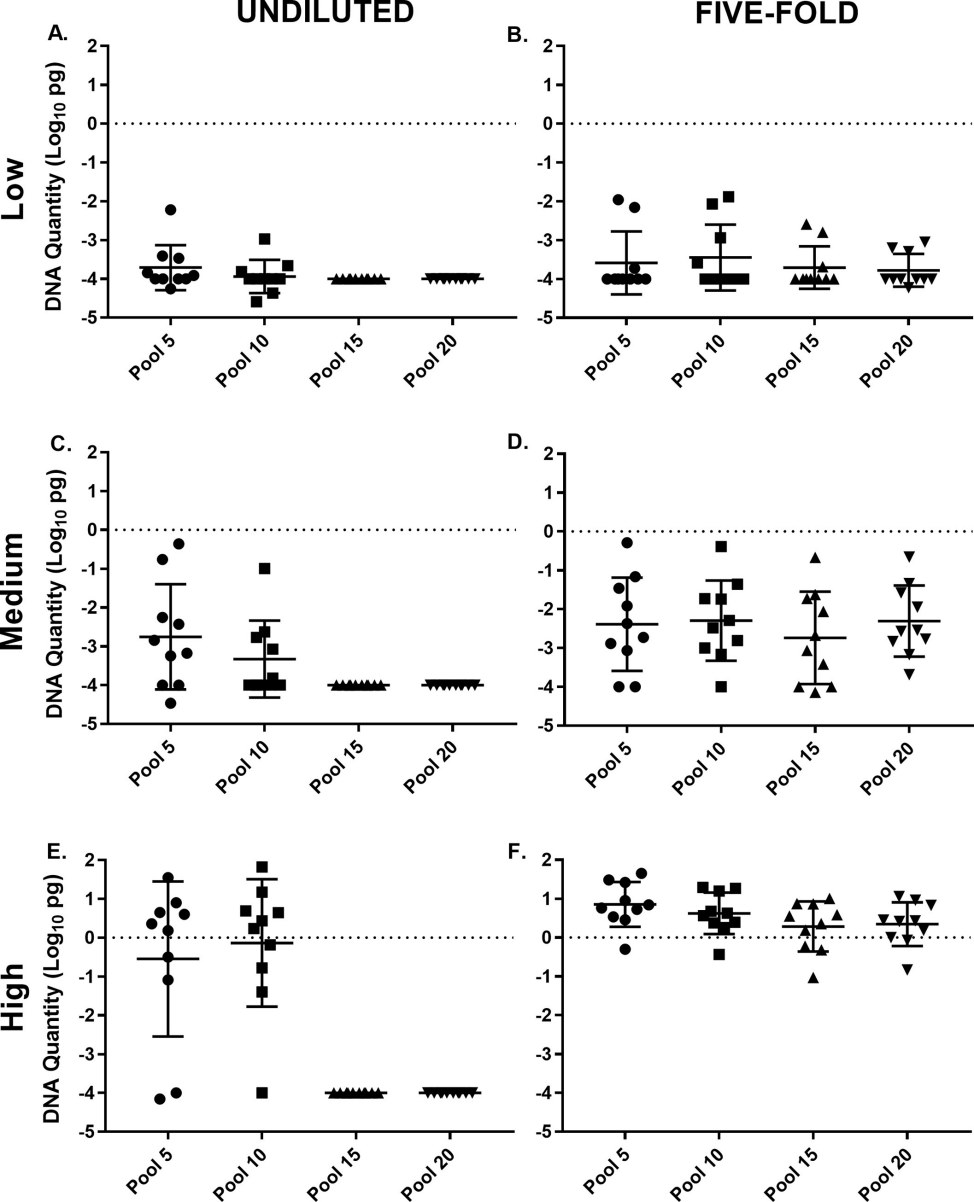
Scatter plots of the log transformed *Mycobacterium avium* subsp. *paratuberculosis* (*Mptb*) mean DNA (± SD) quantities detected in qPCR for each pool size. The undiluted DNA extract of the Low, Medium, and High *Mptb* shedding group (A, C, and E). The five-fold dilution of the undiluted DNA extract of the Low, Medium and High *Mptb* shedding group (B, D, and F). Note the lack of DNA detection for the undiluted DNA extract at pooling rates of 15 and 20 for the Low, Medium and High *Mptb* DNA shedding groups.

For the high shedders group, only pool sizes 5 and 10 had any positive results detected in the undiluted DNA samples (Fig 2). A five-fold dilution of the undiluted DNA extract led to the detection of all (100%) of the samples for all pool sizes. More specifically, pool size 5 and 10 had an additional two positive samples and pool size 15 and 20 went from 0% (0/10) to 100% (10/10) positive, when the undiluted DNA extract was diluted five-fold, which suggests that inhibitors may be relieved with the process of dilution.

No additional positive samples were detected with a 25-fold dilution, compared to the undiluted and five-fold DNA dilution, for the majority of the pool sizes in the different groups (Table 2). Rather, a dilution effect occurred, as more results were detected positive with the five-fold dilution, compared to the 25-fold dilution. However, for pool size 20 in the low prevalence group, the 25-fold dilution unexpectedly led to the detection of two positive results that were not detected in the undiluted and five-fold dilution.

### Sensitivity and specificity

The diagnostic sensitivity was calculated based on the proportion of test positive results within the given pool size (Table 3), relative to individual faecal qPCR. A pool size of 10 was found to have the highest sensitivity for the low and medium group, at 30% (95% CI: 7–65%) and 90% (95% CI: 56–100%), respectively (Table 2). All pool sizes were suitable for detection of samples for the high *Mptb* DNA group with a consistent sensitivity of 100%. Comparing the sensitivity of individual pool sizes for all dilutions, a pool size of 10 had the greatest sensitivity at 73% (95% CI: 54–88), compared to both pool sizes 5 and 20 at 63% (95% CI: 44–80%) and pool size 15 at 57% (95% CI: 37–80%) (Table 3)

**Table 3:** Estimated sensitivity for the pool sizes of faecal samples with low, medium and high quantities of *Mptb* DNA, relative to individual faecal qPCR.

*Mycobacterium avium* subsp. *paratuberculosis* faecal shedding level	Pool size	Sensitivity (Number of positive/total)	95% CI*
**Low**	5	0.20 (2/10)	0.03–0.56
	10	0.30 (3/10)	0.07–0.65
	15	0.20 (2/10)	0.03–0.56
	20	0.20 (2/10)	0.03–0.56
**Medium**	5	0.70 (7/10)	0.35–0.93
	10	0.90 (9/10)	0.56–1.00
	15	0.50 (5/10)	0.19–0.81
	20	0.70 (7/10)	0.35–0.93
**High**	5	1.00 (10/10)	0.69–1.00
	10	1.00 (10/10)	0.69–1.00
	15	1.00 (10/10)	0.69–1.00
	20	1.00 (10/10)	0.69–1.00
**All shedding levels**	5	0.63 (19/30)	0.44–0.80
	10	0.73 (22/30)	0.54–0.88
	15	0.57 (17/30)	0.37–0.80
	20	0.63 (19/30)	0.44–0.80

*CI: Confidence interval

Pool size, *Mptb* shedding level and their interaction were tested in generalised linear mixed models. The interaction was not significant (*P* = 0.96) and hence removed. As expected, the variable *Mptb* shedding level was significant (*P* = 0.0003) indicating that the probability of detection increased with *Mptb* shedding. However, the pool size variable was not statistically significant after adjusting for the *Mptb* shedding level variable (*P* = 0.22) or without it (*P* = 0.27).

The diagnostic specificity was calculated based on the proportion of ‘negative’ pools that tested negative for each pool size, relative to individual faecal qPCR. The pooled testing demonstrated 100% specificity, with all negative pools having negative test results in the qPCR (40/40), independent of the pool size.

### Effects of additional DNA dilutions on the results for a pool size of 10

**Fig 3:**
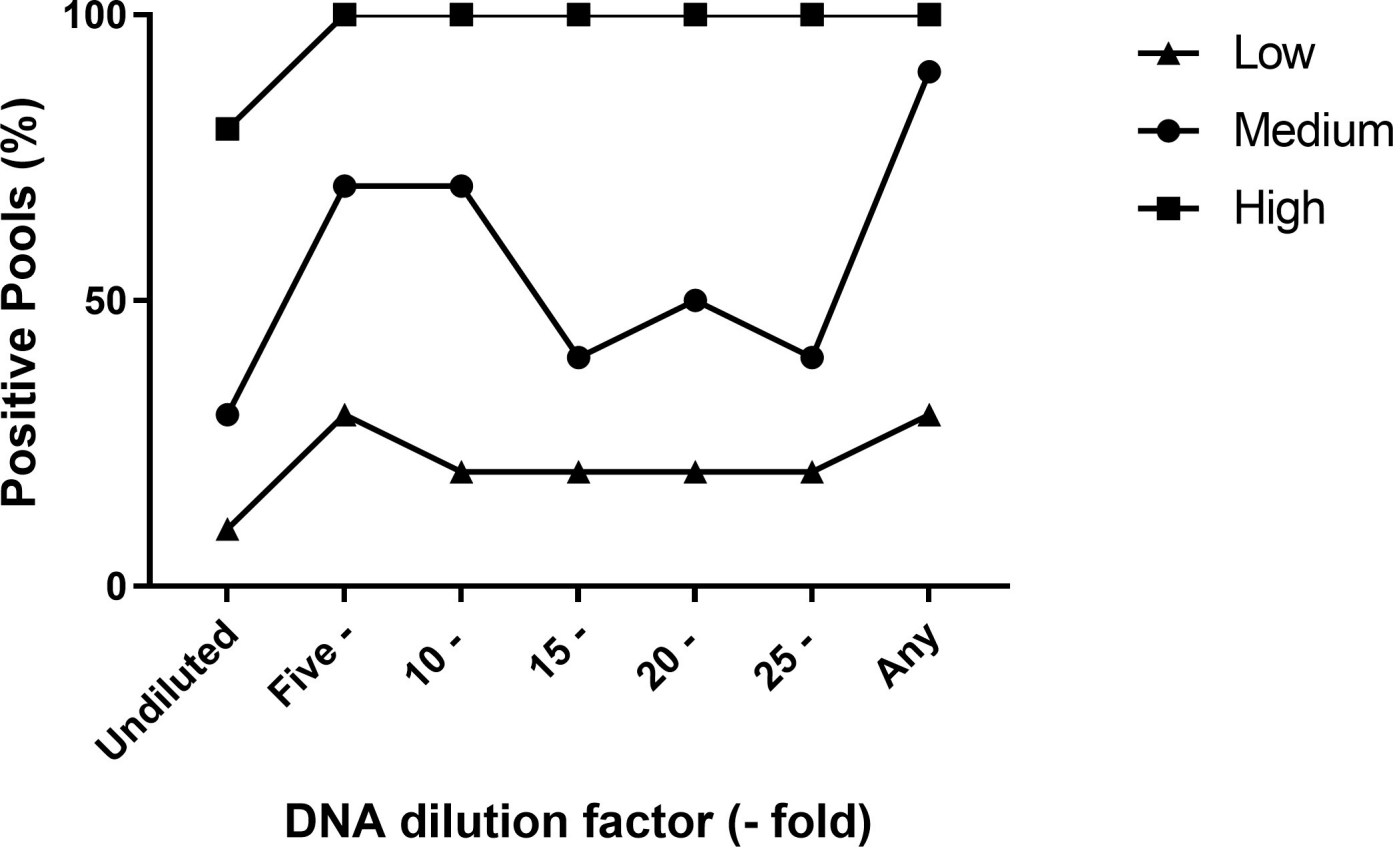
The DNA dilution factors applied to the pool size of 10 for different levels of *Mptb* faecal shedding.

The DNA extracts for samples prepared at a pool size of 10 was further diluted (10-, 15- and 20- fold) to determine whether the optimal dilution rate would relieve PCR inhibitors. For the low shedding group, the five-fold dilution had the most positive results at 30% (Fig 3). For the medium level shedding group, both the five-and ten-fold DNA dilution enabled 70% of the samples to be detected positive. The dilution of the undiluted DNA extract for the high shedding group resulted in all (100%) samples testing positive, regardless of the dilution factor.

**Fig 4:**
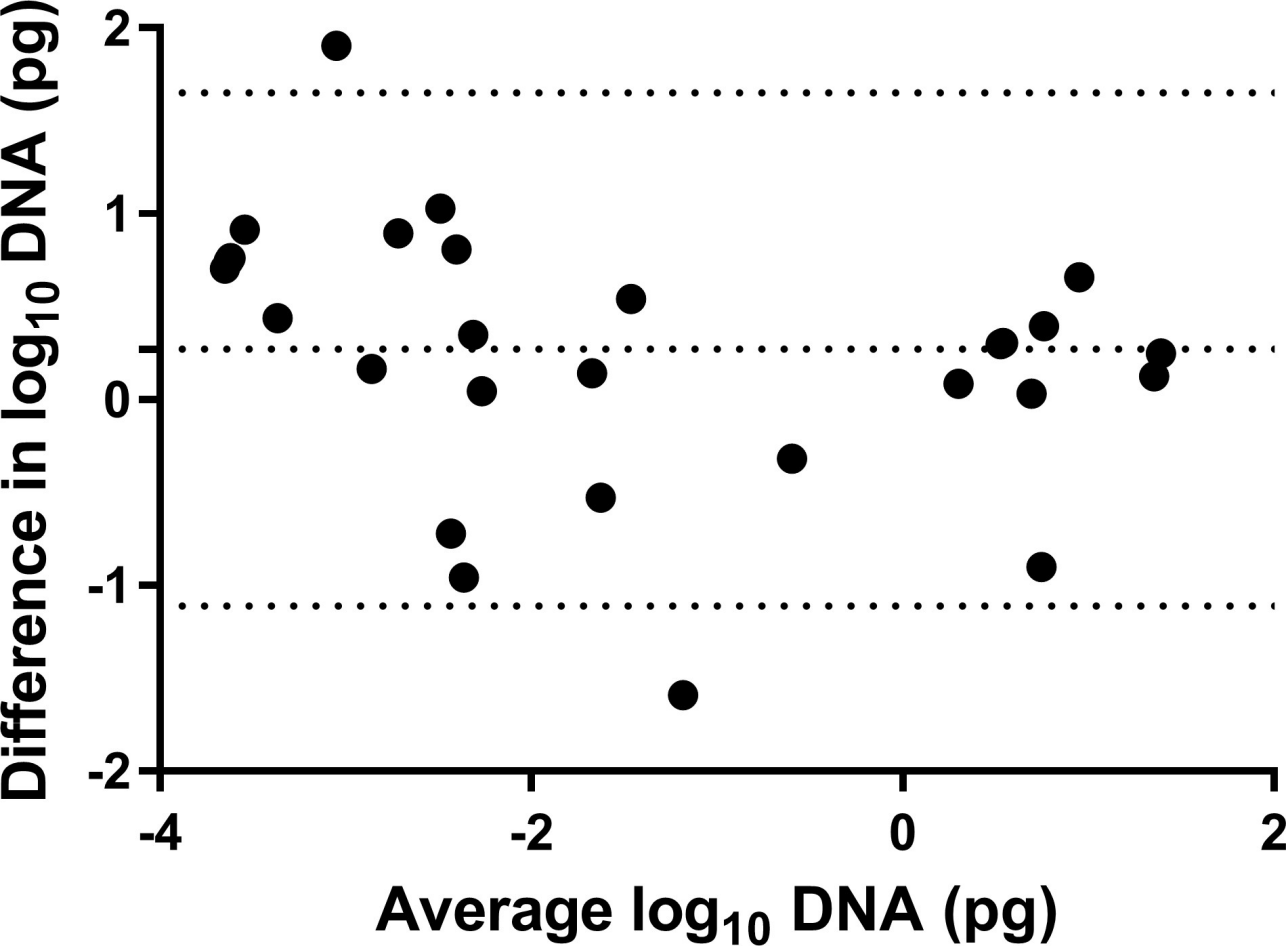
The Bland Altman plot compares the average log transformed of the original DNA quantities of positive individual animal included in the pool against the five-fold dilution of the pool size of 10. The dotted middle line and two-outer dotted line represents the 95% region of agreement.

The Bland Altman plot demonstrates the log_10_ difference between the original DNA quantities against the average DNA quantity of pool size 10 in a five-fold dilution (Fig 4). The overall difference is positive (0.273) as the graph demonstrates two outliers, with most of the DNA samples within the 95% agreement limits (-1.108, 1.653). The positive mean difference suggests that there was a lower DNA quantity detected in the five-fold dilution of pool size 10, than in the original DNA quantity of individual animals, as would be expected. From the graph, it is evident that the differences are evenly scattered, with negligible pattern or trend that follows an increase in average DNA quantity.

The results of the faecal qPCR for the pool size of 10 were comparable to the individual qPCR results, relative to faecal culture outcomes. The decreased of DNA quantity detectable from the process of diluting the pool size of 10 was most prominent in the low and medium groups for the samples that were culture-negative (n = 7 low and n = 2 medium samples). This contrasts to the culture positive samples within these groups; as all of these samples (n = 3 low and n = 8 medium) were test-positive in the pooled faecal qPCR and further dilution of the DNA extract demonstrated a relatively stable decline (S1 Fig).

## Discussion

The objective of this study was to investigate alternative pooling strategies (pool sizes of 10, 15 and 20) and compare them to the current pool size of 5 recommended for the direct faecal qPCR of pooled cattle samples applied in the J-BAS screening test. Pools of JD positive faecal samples with low, medium and high levels of *Mptb* DNA were used to mimic the collection of faecal samples from low, medium and high faecal shedding cattle. This study found that increasing the pool size could produce results of similar sensitivity and specificity to the current pool size used.

The pool size of 10 performed similar to the pool size of 5 for test sensitivity. It achieved an overall sensitivity of 73% (95% CI: 54–88%), compared to a sensitivity of 63% (95% CI: 44%– 88%) for the pool size of 5 for the different levels of *Mptb* faecal shedding. However, further increases in pool size tended to decrease the overall sensitivity, with pool sizes of 15 and 20 having a test sensitivity of 57% (95% CI: 37–80%) and 63% (95% CI: 44–80%), respectively. These results are in agreement with a recent study in dairy cattle using PFC and IS*900* PCR, which demonstrated a similar test sensitivity of 78% for the pool size of 10 [25]. In addition, they similarly found that increasing the pool size to 20 or 30 resulted in decreased positive results detected, which was especially evident for the low *Mptb* shedding animals [25]. Contrastingly, a recent study on PFC demonstrated that the pool size of 5 had the highest sensitivity at 67%, compared to the pool size of 10 at 59%. Despite this higher sensitivity, there was no significant difference between the two pool sizes, which is similar to our study [12]. From previous research on PFC and our study, we can be confident that the pool size of 10 can perform as well as a pool size of 5, if not better.

The choice of pool size may depend on the amount of *Mptb* DNA present in the faecal sample. In this study, the high *Mptb* shedding group demonstrated 100% sensitivity with all pool sizes, whereas the proportion of positive pools for the medium and low group differed between the pool sizes. For both the low and medium *Mptb* faecal shedding levels, pool size 10 performed better than both a lower pool size of 5 and higher pool sizes of 15 and 20. Our results are partly in agreement with a previous study in which low prevalence herds were better detected in smaller pool sizes for PFC, compared to larger pool sizes [12, 16]. Moreover, a separate study demonstrated that the sensitivity of the detection of moderate to heavy shedders for the pool sizes of 5 and 10 was higher, than the lower shedding group for the same pool sizes [15]. A Monte Carlo simulation also demonstrated that the pool size of 10 or 20 is preferred, when the DNA quantity levels ranged from 0.01 to 0.1 pg, compared to a pool size of 50 [14]. The reason for the poor detection of low *Mptb* DNA quantity in larger pool sizes may be due to the ‘dilution’ effect [13, 31]. This implies that the already low quantity of *Mptb* DNA in the low shedding group may become undetectable when pooled at a larger pool size. This can be demonstrated with the reduction of colony forming units (CFU) of *Mptb* per gram, to below the detection threshold in PFC when a pool size of 5 is used [13]. Therefore, a pool size of 10 has been shown to be able to detect the greatest number of positive results from a range of *Mptb* faecal shedding levels.

The results in this study demonstrated 100% specificity for all the different pool sizes and dilution series. This specificity level is in agreement with a previous study, which demonstrated that non-exposed *Mptb* cattle had a high specificity of 99.6% with the HT-J qPCR, relative to liquid culture [9]. Our results demonstrate that the direct faecal HT-J qPCR when used with pooling, is a robust test with high specificity. The major advantage of a highly specific test is that producers would be able to confidently test their herds for JD with false-positive results unlikely to occur. The regular occurrence of false positive results when used with previous diagnostic assays for JD testing had resulted in producers ‘opting out’ of the control programs, due to the psychological turmoil and financial stresses associated with false positives [32].

Although pooling is economically beneficial, it does seem to exacerbate the effect of PCR inhibition. The pooling and homogenisation process of the faecal samples may result in the unexpected release of compounds from the heterogeneous distribution of microorganisms found in the diet of ruminants. These compounds may hinder PCR amplification and ultimately lead to inhibition. These potential contaminants may include polysaccharides, humic acid, phytic acid, phenols, urea and polycyclic aromatic hydrocarbons [22, 33, 34]. A possible hypothesis is the homogenisation process involved with the crushing of complex plant debris that are normally allowed to settle by gravity in the initial sedimentation step of the individual HT-J qPCR method; this would require investigation of the individual inhibitory substances to be proven. In this study, inhibition was evident in the pools of all *Mptb* shedding levels, with fewer positive test results for the undiluted DNA compared to a five-fold dilution of the DNA extract. PCR inhibition has also been reported previously for the qPCR methodology used in this investigation; a previous study identified that undiluted DNA extracts of individual cattle faecal samples had lower sensitivity (55%), compared to the five-fold dilution of the undiluted DNA extract, with a sensitivity of 76% [22]. In this study, we found that PCR inhibition occurred mainly in the larger pool sizes of 15 or 20 for all *Mptb* shedding levels in the undiluted DNA extract. To our knowledge, this is the first study to demonstrate that increasing the pool size of beef cattle faeces leads to an increase in the severity of PCR inhibition.

Our results indicate that PCR inhibition can be relieved with the dilution of the DNA extract, a common non-specific method used to mitigate PCR inhibitors [19, 22]. The five-fold dilution of the DNA extract resulted in a 91% increase in positive detection of the diluted pools, compared to the undiluted DNA. This demonstrates that a simple non-specific five-fold dilution has the ability to relieve PCR inhibition and lead to a greater positive pool detection. However, diluting the DNA extract too much may reduce the ability to detect the target amplicon in the sample [19, 35]. For instance, the 25-fold dilution conducted in this study led to a decreased number of positive samples being detected in the low and medium *Mptb* faecal shedding group. This decrease suggests that further dilution may have reduced the amount of *Mptb* DNA available in the aliquot, similar to the compromised qPCR sensitivity found when DNA dilution was used to detect *Enterococcus* [19].

Determining the optimal dilution factor is vital to successfully mitigating PCR inhibitors. The further dilution of pool size of 10 for the high *Mptb* shedding group demonstrated 100% sensitivity for all dilution factor tested. The medium shedding group had 70% of the pools detected positive for both the five- and 10-fold dilution; however, a decrease in positive pools was detected when the dilution factor exceeded 10-fold. Similarly, the five-fold dilution was the optimal dilution factor for the low *Mptb* DNA group, as additional dilutions also decreased the proportion of positive pools detected. These results demonstrate that the five-fold dilution is the optimal dilution factor, as there are no additional benefits with the further dilution of the undiluted DNA extract. Our results agree with a previous recommendation of conducting both the undiluted and five-fold dilution of DNA samples to reduce the possibility of diluting and reanalysing the sample, post inhibition [19]. Therefore, we recommend using a five-fold dilution to relieve inhibition in pooled samples.

The pool size of 10 performed optimally for the testing of JD in beef cattle. This pool size had the greatest sensitivity for all *Mptb* faecal shedding levels tested. Although the sensitivity for the pool size of 10 was maximum, it was still not statistically significantly different from the sensitivity achieved for the other pool sizes, suggesting that a higher sensitivity achieved for the pool size of 10 could be a random variation. However, it also means that the sensitivity for this pool size was at least as high as that for the pool size of 5. The high specificity provides a high positive predictive value, synonymous to a high level of confidence that a positive pool would include a truly infected animal. These results are in agreement with a recent study, which modelled the cost-effectiveness of various testing methods in a national JD surveillance program for Irish dairy herds [36]. They demonstrated that pooled faecal testing gave the highest confidence of freedom from disease compared to other herd-level diagnostic tests including serology, bulk milk tank and environmental testing. This study considered faecal culture as interchangeable with PCR testing for pooled faecal samples, based on a previous study that found negligible difference between the sensitivities of the two tests [37]. However, PCR may have the ability to detect additional positives due to the “pass-through” phenomena, where an uninfected animal may have passively ingested *Mptb* and was undetected by culture [38, 39].

A change in testing regime from a pool size of 5 currently used to a pool size of 10 can nearly halve the cost of testing for the beef industry whilst maintaining sensitivity and specificity. For the Sample test, the testing of 300 animals with a pool size of 10 would only require 30 pools to be created and tested, rather than the original 60 pools associated with a pool size of 5. However, for the Check test, the beef industry could either (a) halve the cost and maintain the same confidence of disease freedom by testing the sample size of 50 (i.e. testing five pools of 10 animals each instead of 10 pools of five animal each) or (b) substantially increase the confidence of disease freedom and herd sensitivity, while maintaining the same cost with the current testing regime. This involves testing 10 pools of 10, which increases the sample size to 100. However, the direct effects on confidence of disease freedom and herd sensitivity are influenced by the farms prevalence level and this aspect was not within the scope of this paper. Moreover, it is important to note that the cost will not be halved as additional processing steps may increase the laboratory costs. These potential scenarios demonstrates the cost-effectiveness of the pool size of 10 for J-BAS assurance testing, as it reduces the cost of testing and increases the level of producer confidence.

Further evaluation of an increase in pool size could be conducted using simulation modelling by creating real-life farming scenarios for herd level testing [12, 16]. Factors that may affect the herd sensitivity and overall feasibility of using a larger pool size include herd size, disease prevalence levels, disease state and the level of faecal *Mptb* shedding by animals within the herd [16, 40]. In this experimental study, the pooling ratio of the inclusion of one positive animal for every nine negative animals for the pool size of 10, mimics a prevalence of 10%, which does not represent the correct prevalence of *Mptb* infection level encountered in Australian beef herds. To account for real-life prevalence values, simulation modelling would enable sensitivity estimation at different prevalence levels for the different pool sizes. Moreover, the sensitivity of the pool size of 10, 15 or 20, is yet to be explored in any simulation modelling studies, compared to a pool size of 5, which is the most commonly reported pool size [36]. Pooled faecal testing was shown to have the highest sensitivity for herd level testing of Irish dairy herds, however it was one of the most costly methods [36]. Estimates using different pooling rates may mean the cost is more in-line with other diagnostic test options.

Some limitations of this study include the process of pooling and blending steps, which may result in an uneven distribution of *Mptb* in the sample and thus affect the quantity of bacteria present in the aliquot [15, 41]. The use of archived faecal samples with the process of freeze–thawing may have compromised the DNA integrity, and ultimately affected the detection of some of the samples with *Mptb* DNA quantities. This could have been avoided with the collection and immediate processing of fresh faecal samples. A further limitation was the selection of samples from a limited number of herds, as well as the use of two bulk negative sample for pooling, which may have led to the presence of PCR inhibition due to the similarity in diets. The limited number of animals from different herds was used to reduce the degree of experimental variation in the study, which enabled us to focus on the positive individual animals in the respective pools [18, 26, 40]. However, validation of the presence of inhibitory components in the faeces of animals from different herds in the field is required.

An alternative approach involving pooling DNA extracts rather than faeces [42], may reduce the incidence or effect of PCR inhibition, however, this would substantially increase the cost of testing due to the need for individual DNA extractions to be performed. The single replicate of the 25-fold dilution led to the unexpected occurrence of two positive results for the pool size of 20 in the low *Mptb* DNA group; these positive results may have been due to a stochastic effect on the presence of the target amplicon in the small aliquot sampled for testing by the qPCR [9, 43]. Conducting the 25-fold dilution qPCR in duplicates may have enabled clarity regarding this, as the criteria for a positive test required all replicates to be positive.

This study had a number of strengths. It used a validated diagnostic protocol to recommend an optimal pool size suitable for JD testing. The positive samples selected were from three different *Mptb* faecal shedding levels commonly encountered in the field. The processing of the negative samples in a Physical Containment 2 (PC2) laboratory prior to pooling with the positive samples ensured that the risk of cross contamination was minimised. The PCR inhibition encountered in this study was rectified with a non-specific dilution approach to provide an appropriate recommendation of pool size and dilution factor for beef cattle faecal samples. These aspects provide confidence that the pool size of 10 is the optimal pool size for the HT-J qPCR testing for J-BAS.

In Australia, farmers obtain a J-BAS score to determine the JD infection status of their herd. The costs associated with obtaining and maintaining a J-BAS score can be substantial due to stringent testing requirements associated with the Sample and Check test. This study demonstrated that a pool size of 10 could replace the current pool size of 5, based on its performance for both test sensitivity and specificity. The pool size of 10 for the different levels of *Mptb* faecal shedding demonstrated a similar, if not better performance than the pool size of 5, when the undiluted DNA extract was diluted five-fold to alleviate PCR inhibitors. Despite strong experimental evidence that the pool size of 10 performed optimally compared to the other pool sizes, it is still vital that simulation modelling be conducted with input variables representing real-life Australian farming scenarios, prior to the legislative implementation of our recommended pool size. Simulation modelling would not only provide better estimates of sensitivity for each pool size, but also provide cost-effective suggestions that could not be calculated from experimental studies alone. Therefore, these results suggest that an alternative pool size can be applied to J-BAS testing, in order for Australian beef producers to reduce costs, maximise the number of positive animals detected and accurately determine JD herd prevalence levels.

## Supporting information

S1 Fig The HT-J PCR results of the dilution series for the pool size of 10. The DNA quantities detected for the different dilutions of pool size 10 were compared to the individual faecal qPCR and sub grouped according to the culture status (‘Indiv. Undiluted). The DNA quantities of the individual faecal qPCR was log-transformed and compared to the different diluted DNA quantities (five-, 10-, 15-, 20- and 25- fold dilutions of the undiluted DNA extract). The culture results from the previous HT-J testing for the different levels of *Mptb* shedding group were categorised into culture positive and negative, with averages and standard deviations plotted. The red line distinguishes the positive and negative cut point.
(TIF)
