# Supplementary figures and images for: Determining an optimal pool size for testing beef herds for Johne’s disease in Australia
Source: PLoS One. 2019 Nov 20;14(11):e0225524. doi: 10.1371/journal.pone.0225524 (PMC6867630; doi:10.1371/journal.pone.0225524)

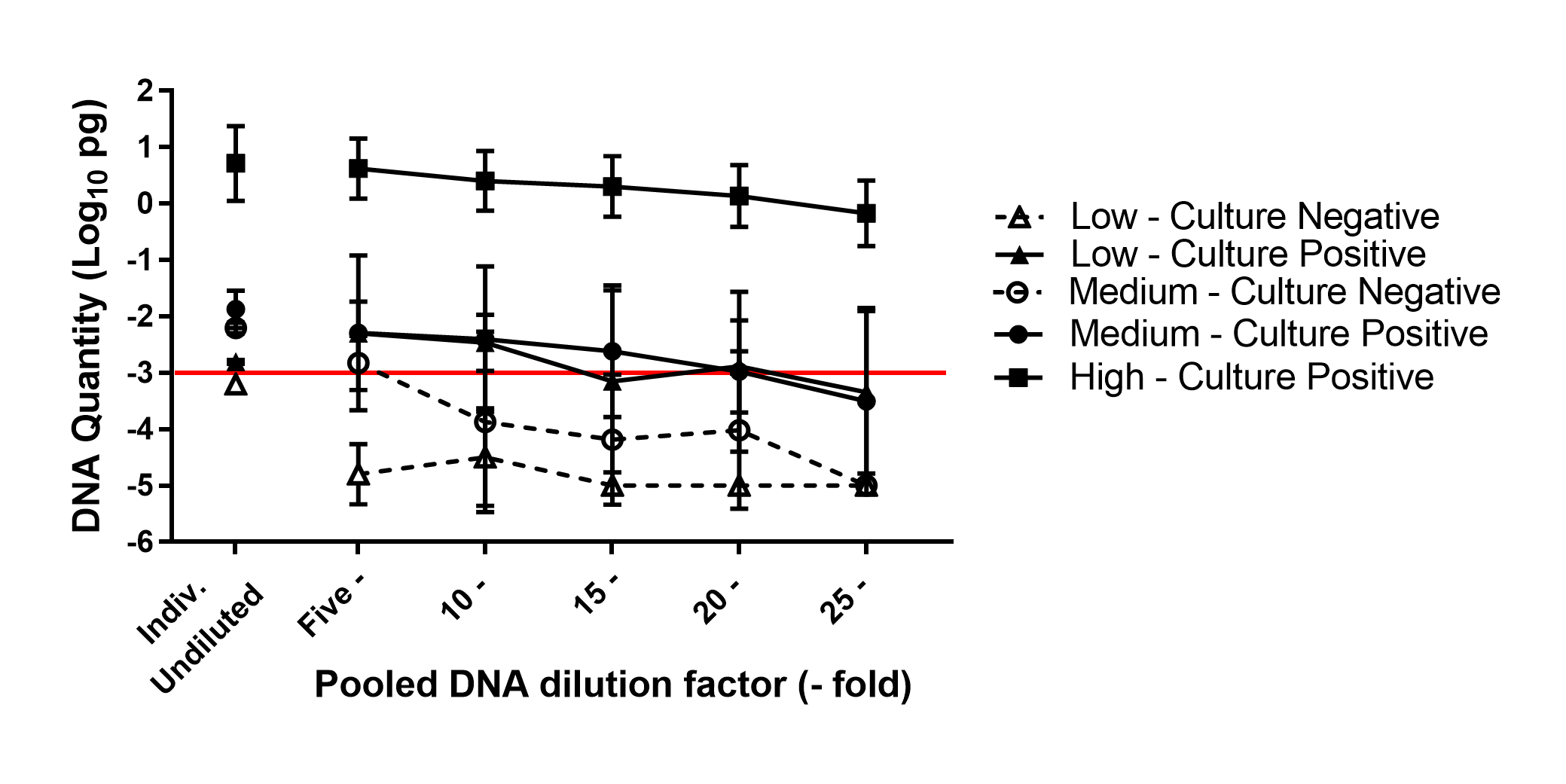

Supplement: S1 Fig — The DNA quantities detected for the different dilutions of pool size 10 were compared to the individual faecal qPCR and sub grouped according to the culture status (‘Indiv. Undiluted). The DNA quantities of the individual faecal qPCR was log-transformed and compared to the different diluted DNA quantities (five-, 10-, 15-, 20- and 25- fold dilutions of the undiluted DNA extract). The culture results from the previous HT-J testing for the different levels of Mptb shedding group were categorised into culture positive and negative, with averages and standard deviations plotted. The red line distinguishes the positive and negative cut point. (TIF) [file pone.0225524.s001.tif]
